# Editorial: Pathways to mental health resilience in emergency personnel: protective strategies and occupational challenges

**DOI:** 10.3389/fpsyt.2026.1967089

**Published:** 2026-08-25

**Authors:** Ulrich Wesemann, Hubertus Himmerich

**Affiliations:** 1 Department of Psychiatry, Psychotherapy and Psychotraumatology, Bundeswehrkrankenhaus Berlin, Berlin, Germany; 2 Department of Psychiatry and Psychotherapy, Charité, Berlin, Germany; 3 Department of Psychological Medicine, Institute of Psychiatry, Psychology and Neuroscience (IoPPN), King’s College London, London, United Kingdom

**Keywords:** emergency responder, firefighter, mental health, military personnel, police officer, prevention, PTSD - posttraumatic stress disorder, resilience

## Introduction

Emergency personnel are typically recruited from the general population. While initial selection processes can result in minor variations in personality traits or resilience, these workers generally share similar characteristics with their working-age peers. Differences in vulnerability to mental health challenges are primarily driven by external risk factors—particularly because military and operational forces frequently carry out their duties in unpredictable, high-pressure environments defined by volatility, uncertainty, complexity, and ambiguity (VUCA) (Mair et al.). Because of these demanding working conditions, mental health challenges are widespread and represent a significant occupational risk (1). Whether working in military operations (2), firefighting (3), policing (4), emergency medicine and nursing (5), humanitarian aid (6), healthcare workers (7), or forensic investigation (Hong et al.), these professionals face occupational environments that continuously challenge psychological wellbeing. At the same time, they must consistently perform under pressure. Therefore, varying combat experiences or other forms of exposure can have significant effects on coping and mental health (8). However, it is becoming increasingly evident that the manifestations of mental disorders differ significantly across various occupational groups. For instance, problematic anger is frequently observed among military personnel (9) and, at times, among police forces (10). At the same time, there is growing evidence that the decisive factor is not the profession itself, but rather the specific nature of the tasks performed on the ground (11). The effects are not limited to those directly affected but also extend to social relationships with family members and the social environment (12).

This Research Topic comprises 43 articles that illustrate the multidimensional nature of research on emergency personnel. Together, they demonstrate that mental health and quality of life develop through complex interactions between exposure to adversity, coping resources, leadership, organisational climate, training, social connectedness, and evidence-based interventions. Across various industries and regions worldwide, the articles highlight shared factors that either protect or harm mental health, while also pointing out unique challenges specific to different professions.

Recurring topics—such as burnout and work-family conflict—continue to pose a threat to the ability to work. At the same time, professional identity, coping strategies, mindfulness, peer support, and organisational interventions consistently prove to be important protective factors. For this Research Topic, we have also secured a diverse range of contributions based on contemporary research approaches; these include systematic reviews, structural equation modelling, latent profile analysis, qualitative studies, and intervention studies, as well as innovative applications of artificial intelligence and deep learning. To facilitate a better understanding of this diverse body of work, we have categorised the contributions into six thematic areas.

## Theme 1: occupational stress and burnout

This research focus addresses the psychological strain experienced by nursing and emergency medical personnel and its impact on their fitness for duty. As nursing becomes an increasingly academic profession, the number of scientific studies focusing on nurses’ mental health is also growing (13). This is a highly positive development, given that the topic is gaining increasing importance amidst growing demand and limited resources. Across healthcare professions, burnout is viewed not merely as an individual health issue but as a multifaceted organisational challenge affecting staff retention (14), work performance (15), patient safety (16), and the quality of care (17). Various studies on the intention of nursing and emergency personnel to change jobs or leave the profession (16) highlight how frequent work interruptions (Wang & Zhu), excessive workloads (18), occupational stress (19), and emotional exhaustion (20) contribute to this decision. Furthermore, systematic findings regarding the work-life balance of emergency and nursing staff demonstrate that sustainable career paths depend on organisational frameworks that facilitate recovery from chronic occupational stressors (Pryba et al.). Risk factors such as moral distress (Dong et al.), salary dissatisfaction and the challenging work environment are also discussed (Szczupak et al.).

The Research Topic further illustrates that burnout rarely occurs in isolation. It reveals that compassion fatigue, secondary traumatic stress, anxiety symptoms, and poor sleep quality, interact in complex ways and often mutually reinforce one another through reciprocal psychological processes. The conflict between work and family continues to play a special role in this regard (Zhong et al.). Sophisticated analytical approaches—including latent profile analysis (Yao et al.) and structural modelling (Lan et al.)—reveal considerable heterogeneity among emergency personnel. In doing so, they demonstrate that work-related stress is not experienced in the same way by all employees. Such findings support the growing recognition that prevention strategies should be tailored to specific risk profiles rather than relying on unspecific interventions (Bani-Hani et al.).

An innovative aspect of the contributions in this chapter is the identification of modifiable organisational factors capable of reducing work-related stress. These include a healthy work environment, supportive leadership, and collegial relationships, as well as a manageable, predictable workload that allows for autonomy in task execution. Measures promoting work-life balance also play a crucial role in emergency medical services (Pryba et al.). Overall, the studies demonstrate that protecting mental health must not rely solely on strengthening individual resilience. Equally important are organisational cultures that minimise unnecessary stressors while fostering professional engagement. Workplace health management and appropriate measures for reintegration following extended absences also constitute essential organisational interventions (Wang & Zhu). Furthermore, these measures should be tailored to the specific needs of the individuals concerned. Consequently, the findings presented here position burnout prevention as a central pillar of professional resilience across all emergency service professions.

## Theme 2: risk and resilience factors

The second thematic area examines resources that enable emergency personnel to maintain their well-being. Conversely, there are also risk factors that make emergency personnel more vulnerable. Childhood maltreatment is among the significant risk factors; This can foster not only PTSD but also pain disorders—particularly in conjunction with PTSD (21). In this context, resilience is not viewed as an innate or static trait. Consistent with recent research, the studies describe resilience as a dynamic process that can be developed. This process is influenced by the interplay of cognitive, emotional, behavioural, and interpersonal factors. Various groups of emergency personnel were studied: nursing staff, firefighters, emergency medical services personnel, and military personnel. Resilience was found to be closely linked to professional identity, adaptive coping strategies, successful emotion regulation, social support, and psychological flexibility.

Several studies examine mechanisms of resilience. Professional identity appears to be associate with reduced anxiety and better mental adjustment. Sleep quality emerged as a mediator between job demands and mental health (Cao et al.). On the other hand, even among nursing staff with an intact professional identity, pronounced sleep disturbances were identified as a key factor associated with higher levels of anxiety symptoms (Liao et al.). Other studies highlight the importance of cognitive appraisal, proactive coping, emotion regulation, sport-related self-efficacy, and psychological capital as resilience factors. Of particular interest are the appraisal of highly stressful situations, the associated emotional reactions, and emotion regulation. This could lead to a more holistic understanding of the underlying cognitive-emotional processes and, consequently, to better preparation (Poless et al.). Moral resilience, moral courage, and moral sensitivity are also becoming increasingly important topics in contemporary research (Xiang et al.). In this context, it has also been shown that perceived social support strengthens psychological resilience (Zhao et al.). Taken together, these findings support transactional stress models, according to which individual interpretation and adaptive coping strategies significantly influence the psychological consequences of exposure to stressful events (22).

This area also highlights that resilience is more than just the avoidance of mental health symptoms. Positive adaptation encompasses post-traumatic growth (Prykhodko et al.), sustained professional engagement (Khalil et al.), flourishing in the workplace, and continued professional performance (Bosomtwe and Nubuor) despite adverse circumstances. Mindfulness-based interventions and structured resilience programs provide promising evidence that protective competencies can be consciously strengthened through targeted training, rather than being viewed as traits.

Taken together, these studies demonstrate the benefits of positive psychology strategies. They acknowledge significant psychological challenges as occupational risks to mental fitness. However, preparatory training prior to deployment and the optimisation of the work environment can have a positive effect (Borck et al.; Dong et al.). At the same time, however, they focus on modifiable protective factors capable of enhancing both individual well-being and organisational performance. As in most other areas, tailored measures are considered more helpful here than a one-size-fits-all approach (Chen et al.). Future resilience research will benefit from the integration of biological, psychological, social, and organisational perspectives. This enables a better understanding of the interplay between protective mechanisms over the course of emergency responders’ careers.

## Theme 3: occupation-specific factors

Although professions in emergency services and crisis response entail similar psychological demands, resilience can manifest differently depending on the specific operational contexts of these roles. For instance, suicide is a common phenomenon in a military context—such as in connection with PTSD or depression (23, 24). Studies covering emergency medical services, air rescue crews, firefighters, police officers, forensic investigators, correctional officers, and school police officers were included in this chapter. Collectively, they illustrate how professional cultures, stress patterns, organisational structures, and operational tasks influence both psychological risks and protective mechanisms.

Emergency medical services personnel are repeatedly exposed to traumatic events and unpredictable, mission-related stressors. Among individuals in this occupational group aged 35 and older, a negative association is observed between burnout symptoms and the willingness to participate in prevention programs (Austel et al.). Furthermore, studies on air rescue crews highlight that the specific characteristics and intensity of missions shape the psychological strain in a distinctive way. At the same time, the research showed that air rescue crews—regardless of age—rarely avail themselves of aftercare measures following challenging missions (Ziehr et al.). Among forensic investigators, traumatic experiences at the scene during disaster victim identification procedures, as well as adverse working conditions, were linked to probable PTSD (Hong et al.).

Clinical decision-making situations with far-reaching consequences are also an integral part of this profession. A study revealed differences in knowledge and attitudes regarding suicide among healthcare professionals. This provides important insights for the implementation of targeted suicide prevention measures (Kashiwagi et al.). Research findings regarding trauma-focused interventions (Jones et al.), interacting with people with mental illness (Karystianis et al.), organisational and operational stress (Moreno et al., 2026), and risky driving behaviour (Alasqah & Noureldin)-complemented by recommendations for a supportive work environment and targeted strategies for burnout prevention (Okoka et al.)-highlight the cumulative effects of chronic, mission-related strain. At the same time, these insights point to potential avenues for targeted organisational support measures. Consequently, profession-specific screenings and corresponding intervention strategies appear warranted (Rubio Vilalta et al.; Casella et al.). In this context, Forsyth et al. rightly highlight that the emotional support of emergency personnel is a matter of institutional responsibility and a public health concern.

## Theme 4: military personnel and special forces

The concept of resilience is gaining increasing importance in military and humanitarian contexts. This is because personnel in these fields are exposed to danger, uncertainty, and potentially life-threatening situations over extended periods. The contributions collected here expand the understanding of occupational resilience beyond the realm of traditional emergency services. Instead, they focus on the psychological adaptability of military personnel, special forces personnel, humanitarian aid workers, and civilian volunteers in conflict zones. Taken together, these studies demonstrate that resilience is not merely a reaction to traumatic stress. They show that it is a capacity that can be actively fostered before, during, and after a deployment.

Several articles address military operational readiness and personnel selection. They highlight the importance of identifying and fostering psychological traits associated with successful performance in extremely demanding operational environments (da Silva et al.; Mair et al.). Research into personality traits, mental strategies, psychological readiness, and adaptive resilience training suggests that psychological preparation should be viewed as an integral component of operational readiness (Ziehr & Merkt). These studies reinforce the growing recognition that resilience can be systematically strengthened through targeted instruction, realistic training scenarios, and continuous professional development.

Further studies examine psychological adjustment following combat deployments. Research on proactive coping and post-traumatic growth among mobilised military personnel indicates that recovery trajectories are influenced by both individual coping resources and broader social contexts—including family relationships (Prykhodko et al., 2025). Complementing this, a quasi-experimental evaluation of combined trauma therapy and computer-based neurocognitive training for soldiers with post-traumatic stress disorder highlights the potential of innovative therapeutic approaches to improve both psychological symptoms and cognitive functions (Muschner et al.).

The humanitarian dimension of resilience is also given due consideration. Community-based narrative interventions during wartime, the personal experiences of ZAKA volunteers in the context of the Israel-Hamas conflict (Cohen Zada, Cohen Zada), and the collaborative development of peer-support programs for Ukrainian professionals (Prykhodko et al.) in the health and humanitarian sectors underscore the importance of collective resilience. They highlight the significance of shared meaning-making and social connectedness during protracted crises. Rather than focusing solely on individual adaptation, these studies emphasise resilience as a This process emerges through collaboration, mutual support, and culturally sensitive interventions. By incorporating insights from various disciplines, the understanding of psychological resilience is broadened to include a moral perspective, opening up potential applications for emergency personnel (Thompson et al.). Taken together, the contributions demonstrate that resilience in military and humanitarian contexts encompasses preparation, operational performance, recovery, and long-term reintegration. This requires coordinated efforts at the individual, organisational, and societal levels.

## Theme 5: training and prevention

While understanding risk factors remains essential, many contributions in this area of ​​research focus on developing and evaluating interventions to strengthen psychological well-being. The aim is to prevent the onset of severe mental disorders. This preventive approach reflects a significant evolution in occupational health research, shifting the focus from purely reactive treatment to proactive capacity building. Studies across various emergency service professions demonstrate that organisational commitment, structured professional development, and evidence-based training can substantially enhance resilience and foster sustainable emergency responder well-being.

Various interventions aim directly at fostering psychological competencies. These include group programs for emergency responders grounded in trauma-informed educational principles, mindfulness training for intensive and paediatric care staff, resilience-oriented educational initiatives, and structured peer support programs (Welton-Mitchell et al.). Promoting the mental health of emergency personnel requires targeted communication strategies that involve leadership (ODare et al.). Collectively, they seek to strengthen adaptive coping strategies before occupational stress reaches overwhelming levels. All these interventions are based on the understanding that resilience is a learnable skill rather than an immutable personality trait. They thus support modern models of psychological development over the course of a professional career and align with the preceding chapters.

Organisational approaches are receiving equal attention. Contributions are presented on topics such as workplace well-being, communication strategies to raise awareness of mental health among emergency personnel, professional training on handling life-threatening situations (Ziehr & Merkt), and well-being programs for healthcare and humanitarian workers (Welton-Mitchell et al.). Together, they illustrate the growing range of institutional responses to work-related stress. Rather than focusing solely on symptom relief, these initiatives aim to create mentally healthy work environments that foster open communication, mutual support, reflection, and the early identification of stressors.

Leadership, an ethical climate, supervision, a supportive workplace culture, and opportunities for professional development can contribute to maintaining psychological well-being. Furthermore, the results indicate that preventive measures are most effective when embedded within broader organisational structures. Taken together, the contributions presented in this area advocate for a comprehensive prevention model. The aim is to strengthen resilience through coordinated measures that involve individuals, teams, leaders, and institutions. Such an integrated approach combines the fostering of personal coping skills with organisational frameworks.

## Theme 6: innovation and future perspectives

This final thematic area within the research focus reflects the rapidly evolving landscape of research into workplace mental health. It highlights emerging technologies, novel analytical approaches, and innovative perspectives that can positively influence the promotion of resilience among emergency personnel. The contributions demonstrate how advancements in digital technologies, predictive analytics, communication science, and implementation research are expanding the methodological and practical horizons of resilience research.

Latent profile analyses reveal significant heterogeneity regarding resilience, burnout, compassion fatigue, and professional identity. In doing so, they challenge the assumption that emergency responders constitute a psychologically homogeneous population and support the findings from Chapter Three. Such person-centred approaches offer the potential to develop more tailored interventions (Alnazly et al.). Likewise, the use of deep learning techniques underscores the growing importance of artificial intelligence in the early detection or prediction of mental strain and disorders (Jang et al.). Rather than replacing clinical assessment, these technologies can complement existing occupational health programs. This enables earlier detection and more individualised prevention strategies.

Other contributions examine the broader ecosystem in which mental health promotion takes place. Research findings regarding the attitudes of care professionals toward generative artificial intelligence underscore the importance of integrating new technologies into daily workflows. These technologies can serve as both analytical tools and workplace innovations. Ultimately, they can thus influence both professional practice and mental well-being (Alnazly et al.). Investigations evaluating mental health awareness communication materials for first responders further demonstrate that effective resilience promotion depends upon how scientific knowledge is translated into accessible, acceptable, and actionable information. Likewise, studies examining healthcare professionals’ attitudes toward patients at high risk of suicide underscore the continuing importance of education and stigma reduction in strengthening compassionate and evidence-informed practice (Kashiwagi et al.).

Taken together, these contributions demonstrate that future research on workplace resilience will be increasingly interdisciplinary in nature. A contemporary approach combines insights from psychology, implementation science, data science, organisational behaviour, education, and communication. At the same time, the findings consistently underscore a key message of this research focus: technological innovations play a crucial role in this field. They are most effective when embedded in an environment characterised by a supportive organisational culture, ethical leadership, and person-centred approaches to mental health. Future research should therefore aim to link methodological innovations with practical implementation.

This ensures that scientific advances lead to tangible improvements in the well-being and operational readiness of emergency personnel.

## Conclusion

The 43 contributions gathered in this Research Topic offer a broad, contemporary overview of current research on the mental health of emergency personnel. Despite the diversity of professions, continents, methodological approaches, and theoretical perspectives, the articles share a common understanding: Mental health among emergency personnel is a dynamic, multidimensional process. Consequently, pre- and post-deployment support, health-promotion measures, and de-stigmatisation programs must be tailored to the specific target groups. Psychological well-being is shaped not only by exposure to potentially traumatic events but also by organisational culture, the ethical climate, leadership, professional identity, personal life circumstances outside of work, social support, opportunities for recovery, and access to evidence-based interventions.

A key contribution of this Research Topic is that it moves beyond deficit-oriented models of workplace mental health. Nevertheless, burnout, psychological strain, compassion fatigue up to the point of emotional blunting, secondary traumatic stress, and moral injury remain important topics. However, the studies consistently demonstrate that these phenomena are neither inevitable nor irreversible. Protective factors—such as adaptive coping strategies, moral resilience, improved selection, target-group-specific and application-oriented training, professional identity, mindfulness, psychological flexibility, supportive leadership, peer support, and organisational commitment to well-being—can significantly strengthen individual and collective resilience. These findings support a paradigm shift away from crisis management toward comprehensive, targeted prevention, early intervention, and sustainable workforce development. In this context, successful models typically consist of a bundle of coordinated measures spanning the entire career cycle, rather than isolated interventions.

Equally noteworthy is the range of professional groups of emergency responders represented in this Research Topic. Emergency nurses, physicians, paramedics, firefighters, police officers, forensic experts, correctional officers, military personnel, humanitarian aid workers, and volunteers each face specific professional demands while simultaneously sharing common psychological challenges. This diversity underscores the need to combine general workplace mental health principles with measures tailored to the specific circumstances of each professional group. Future health-promotion initiatives should therefore integrate profession-specific insights into broader organisational strategies. In this way, it is possible to strengthen psychological safety, ethical leadership, interdisciplinary collaboration, and the long-term well-being of emergency service personnel.

A wide spectrum of methodological approaches is also represented. The range extends from systematic reviews, qualitative studies, intervention studies, and longitudinal analyses to structural equation modelling, latent profile analysis, and innovative applications of artificial intelligence and predictive analytics. This diversity enriches the evidence base and strengthens increasingly nuanced approaches to understanding psychological adjustment in high-risk professions.

Taken together, the studies presented in this Research Topic offer compelling arguments for viewing resilience as a shared responsibility rather than merely an individual trait. Safeguarding the mental health of emergency personnel requires coordinated action across multiple levels. They range from individual competencies and supportive teams to responsive organisations and informed policymaking. By integrating psychology with organisational perspectives, educational innovations, ethical considerations, and technological advancements, this Research Topic enriches the field of workplace mental health. It establishes a foundation for future research, the development of interventions, and evidence-based policymaking. Ultimately, the findings from these 43 contributions underscore the Research Topic’s central message: strengthening the mental fitness of emergency personnel is essential. This serves not only to protect those deployed on the front lines but also to ensure the effectiveness, sustainability, and humanity of emergency services worldwide. When emergency personnel are sidelined by mental health issues during crises, the consequences are typically far more severe.

We are thankful to all authors who submitted excellent manuscripts and to the reviewers who gave useful feedback and thus helped to improve this Research Topic.
